# Draft Genome and Biological Characteristics of *Fusarium solani* and *Fusarium oxysporum* Causing Black Rot in *Gastrodia elata*

**DOI:** 10.3390/ijms24054545

**Published:** 2023-02-25

**Authors:** Jinshao Li, Ke He, Qian Zhang, Xiaoyi Wu, Zhong Li, Xuejun Pan, Yong Wang, Cheng Li, Manman Zhang

**Affiliations:** 1 Key Laboratory of Agricultural Microbiology of Guizhou Province, College of Agriculture, Guizhou University, Guiyang 550025, China; 2 State Key Laboratory for Biology of Plant Diseases and Insect Pests, Institute of Plant Protection, Chinese Academy of Agricultural Sciences, Beijing 100193, China

**Keywords:** *Gastrodia elata* brown rot, *Fusarium oxysporum*, *Fusarium solani*, biological characteristics, whole-genome sequencing, comparative genomics

## Abstract

*Gastrodia elata* is a valuable traditional Chinese medicinal plant. However, *G. elata* crops are affected by major diseases, such as brown rot. Previous studies have shown that brown rot is caused by *Fusarium oxysporum* and *F. solani*. To further understand the disease, we studied the biological and genome characteristics of these pathogenic fungi. Here, we found that the optimum growth temperature and pH of *F. oxysporum* (strain QK8) and *F. solani* (strain SX13) were 28 °C and pH 7, and 30 °C and pH 9, respectively. An indoor virulence test showed that oxime tebuconazole, tebuconazole, and tetramycin had significant bacteriostatic effects on the two *Fusarium* species. The genomes of QK8 and SX13 were assembled, and it was found that there was a certain gap in the size of the two fungi. The size of strain QK8 was 51,204,719 bp and that of strain SX13 was 55,171,989 bp. Afterwards, through phylogenetic analysis, it was found that strain QK8 was closely related to *F. oxysporum*, while strain SX13 was closely related to *F. solani*. Compared with the published whole-genome data for these two *Fusarium* strains, the genome information obtained here is more complete; the assembly and splicing reach the chromosome level. The biological characteristics and genomic information we provide here lay the foundation for further research on *G. elata* brown rot.

## 1. Introduction

*G. elata* is a valuable medicinal plant in China with great significance in agriculture and medicine. It has great potential for the treatment of various diseases. With the rapid development of *G. elata* cultivation, its planting area continues to expand, which is followed by the outbreak of major diseases. Brown rot is the most common and harmful disease in the production of *G. elata*. However, due to the lack of systematic understanding of the disease and its pathogenic mechanism, no effective means for disease prevention and control exist. Therefore, we systematically studied the strains causing brown rot, hoping to find key information for the prevention and control of the disease. First described in the early 19th century, the *Fusarium* genus was classified by Wollenweber and Reinking in 1935 based on morphological differences [1]. *Fusarium oxysporum* is well known for invading its host to induce pathological changes; its induced diseases include *Fusarium* wilt and root rot [2], which cause crop losses [3]. *Fusarium solani*, first described by von Martius in 1842 as the cause of potato tuber rot, is one of the most common fungi in soil and plant debris [4]. This species also causes disease and crop losses (e.g., in Spanish strawberry) [5]. Because *Fusarium* has a wide range of hosts, it massively impacts the world economy due to the crop losses it brings about. Therefore, it is one of the most studied fungi [6].

**Figure 1:**
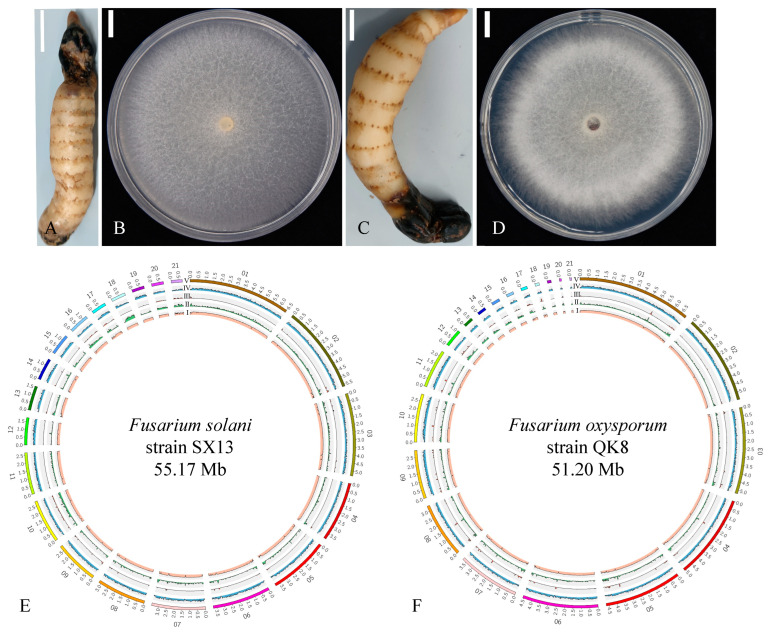
Information on strains SX13 and QK8. (**A**,**C**): The host of strains SX13 and QK8, respectively. (**B**,**D**): Colony morphology of strains SX13 and QK8, respectively. (**E**,**F**): The genome visualization map of strains SX13 and QK8, respectively. I: G+C content; II: repetitive sequence; III: long terminal repeat (LTR); IV: gene density; V: genome fragment size.

*Gastrodia elata* is a perennial heterotrophic herb of the Orchidaceae family, mainly distributed in China, Korea, and India. The species contains organic acids, sterols, and phenolic compounds [7], which have great potential in the treatment of various diseases (e.g., cardiovascular and cerebrovascular diseases) [8]. Gastrodin antifungal proteins (GAFPs or gastrodianins) isolated from *G. elata* show strong antibacterial activity against broad-spectrum fungi [9]. At present, research has shown that *Botrytis cinerea* can cause *G. elata* flower gray mold [10]. Brown rot, a major disease in *G. elata* cultivation, was found in *G. elata* production areas in Dejiang County, Guizhou Province. The damage was severe. Through previous research, we found that the disease was caused by *F. oxysporum* and *F. solani* [11,12]. In these previous articles, we described in detail the pathogenic fungi that caused the disease. Here, we describe the plants of *G. elata* affected by brown rot (Figure 1A,C) and the colony morphology of strains QK8 and SX13 isolated from the brown rot (Figure 1B,D). Some of the parts of *G. elata* infected with brown rot had black or brown lesions, tissue decay, and a pungent odor, and the infection constantly spread to healthy parts, resulting in the whole *G. elata* tuber being affected. Among the two strains isolated from the infected site of *G. elata*, the hyphae of strain QK8 grew outward in a round shape. The hyphae were loose like villi, and the colonies were white at the beginning before gradually turning red. Strain SX13 grew in a round shape close to the culture medium with few and sparse hyphae, white at first before gradually turning gray.

Studying the biological characteristics of fungi can shed light on the colony growth of pathogenic fungi and the yield, germination, and toxicity of conidia [13]. Moreover, such research can reveal the inhibitory effect of fungicides on pathogenic fungi [14] to lay the foundation for disease prevention and control. Genome sequencing provides insights into the evolution of pathogens of phytopathogenic fungi [15]. At present, the genomes of several phytopathogenic fungi are available in public repositories, including *Puccinia striiformis* f. sp. *tritici* [16], *Magnaporthe oryzae* [17], and *B. cinerea* [18]. *F. oxysporum* and *F. solani*, two common phytopathogenic fungi, are consistently being studied. For *F. solani*, three draft genome sequences have been published for strains FSSC 5 MPI-SDFR-AT-0091 [19], JS-169 [20], and CRI 24-3 [21]. Many draft genomes of *F. oxysporum* have been deposited in the NCBI database, including for strain Fo47 [22], *F. oxysporum* [23], and *F. oxysporum* f. sp. *capsici* [24]. Further studies have shown that strain Fo47 can inhibit the growth of various plant pathogens. Although the genomes of the two *Fusarium* strains have been published, the draft genome sequence is not perfect, the level of assembly and splicing is still insufficient, and the genome annotation is not comprehensive. Nonetheless, Jelinski et al. (2017) confirmed the highly dynamic nature of the *F. oxysporum* genome [25]. These studies showed that differences exist in the pathogenicity and other aspects of *F. oxysporum* on different hosts.

Despite losses to *G. elata* crops, current research on *G. elata* brown rot is in its infancy, with studies mainly focusing on the occurrence, pathogenesis, and pathogenicity of the disease. The genome of the pathogenic fungi can shed light on its pathogenesis at the molecular level [26]. Therefore, we sequenced and annotated the whole genomes of *F. oxysporum* and *F. solani* isolated from *G. elata* brown rot. The possible causative genes of *G. elata* brown rot were predicted by the annotation results of various databases. Afterwards, the biological characteristics of *F. oxysporum* and *F. solani* were analyzed, hoping to reveal the influence of ecological factors (such as temperature, pH, and drug resistance) on these two strains. Further study on the pathogenesis of *G. elata* brown rot in the future could be conducted based on our sequencing result and could provide new understanding of the disease.

## 2. Results

### 2.1. Biological Characteristics of the Fusarium Strains

The strain QK8 isolated from the susceptible part of *G. elata* grows round and outward on the PDA medium, and the white colonies are fluffy and raised. At the same time, the strain SX13 also grows round on the PDA medium, but the hyphae are few and sparse; the initial growth is white, and then gradually turns gray. The optimum growth conditions of strain QK8 were as follows: pH 9, 28 °C, fructose as a carbon source, beef extract as a nitrogen source, the NA medium as the base medium, and alternating light and dark conditions (Figures S1–S6). The strain died after 40 min at 60 °C (Figure S7). The optimum growth conditions of strain SX13 were as follows: pH 7, 30 °C, mannitol as a carbon source, sodium nitrate as a nitrogen source, PDA medium as a base medium, and dark conditions (Figures S1–S6). The strain died after 40 min at 65 °C (Figure S8). 

The indoor toxicity tests showed that difenoconazole (EC_50_, 0.5910 μg/mL), oxime tebuconazole (EC_50_, 0.6058 μg/mL), tebuconazole (EC_50_, 0.6709 μg/mL), and tetramycin (EC_50_, 0.7220 μg/mL) had better bacteriostatic effects on strain QK8, followed by thiram·carbendazim (EC_50_, 1.3088 μg/mL), myclobutanil (EC_50_, 2.2208 μg/mL), imazalil (EC_50_, 2.3077 μg/mL), and shenzinmycin (EC_50_, 2.4054 μg/mL). The antifungal effect of the other agents was not ideal. For example, the antifungal effect of pyraclostrobin was very poor, and the EC50 was as high as 33,441.59 μg/mL (Table S1). 

For strain SX13, oxime tebuconazole (EC_50_, 0.0836 μg/mL), tebuconazole (EC_50_, 0.1548 μg/mL) and tetramycin (EC_50_, 0.6904 μg/mL) had the best bacteriostatic effects, followed by pentachloronitrobenzene (EC_50_, 1.3453 μg/mL), thiram·carbendazim (EC_50_, 2.5722 μg/mL), and myclobutanil (EC_50_, 4.0920 μg/mL). The germicidal efficacy of the other agents was mediocre and did not reach the ideal range. For example, for pyraclostrobin, the EC_50_ values were as high as 1300.4877 μg/mL (Table S2). In our study, oxime tebuconazole, tebuconazole, and tetramycin had good bacteriostatic effects on strains QK8 and SX13.

### 2.2. Whole-Genome Sequencing

#### 2.2.1. Genome Sequencing, Repeat Analysis, and Assembly

The Illumina and PacBio sequencing platforms were used to sequence the genomes of the two *Fusarium* strains. After quality control, high-quality sequence data of 51,204,719 bp and 55,171,989 bp were obtained for strains QK8 and SX13, respectively, with average lengths of 2,438,320 bp and 2,627,238 bp, respectively. Strains QK8 and SX13 were assembled into 21 contigs with gene coverage of 98.04% and 97.91%. Lens N_50_ was 4,657,513 bp and 3,724,189 bp, N_90_ was 2,323,049 bp and 1,334,751 bp, and G+C content was 47.21% and 50.61%, respectively (Table 1). We visualized the genomic information of strains QK8 and SX13 (Figure 1E,F). The map represents G+C content, repetitive sequences, long terminal repeats (LTRs), gene density, and gene fragment size from the inside to the outside. Therefore, we can clearly understand the differences between the genomes of strain QK8 and SX13 in terms of, for example, genome size and length of contig sequences.

By querying the genome database, it was found that there were significant differences in the reported genome sizes of the two *Fusarium* strains, which was consistent with the results of this study. A comparison of the genome assemblies showed that strain SX13 was larger than strain QK8 in genome size, the predicted number of genes, and genome coverage among other factors.

Compared with that of strain QK8, the genome size of *F. oxysporum* strain 160,527 was slightly smaller (51,139,495 bp), the number of genes was higher (16,536), the GC content was slightly higher (47.78%), the total counts of contig sequences were lower (12), and the N_50_ length was greater (4,884,632 bp). Compared with *F. solani* strain CRI 24-3 and strain SX13, strain CRI 24-3 had a smaller genome (49,564,288 bp), fewer genes (15,374) and counts of contig sequences (12), longer N_50_ length (4,496,268 bp) and more similar GC content (50.7%) to strain SX13.

Gene repeat analysis showed that strains QK8 and SX13 had repeat sequences of 3,702,880 bp and 4,584,422 bp, accounting for 7.23% and 8.31% of their genome, respectively. These repeat sequences mainly include DNA repeats, long interspersed nuclear elements (LINEs), LTRs, and other repeat sequences. Among the repetitive elements of strains QK8 and SX13, DNA repeats accounted for 3.85% and 3.20% of their genomes, respectively, while LINEs accounted for 0.27% and 0.33% and LTRs for 1.74% and 1.55%, respectively. There were Academ and TcMar-Tc1 repeat sequences in the DNA repeats and Copia repeat sequences in the LTRs of strain QK8; these repeat sequences were not found in strain SX13. Short-interspersed elements (SINEs) were found in QK8, accounting for 0.01% of its genome, but not in strain SX13. However, compared with strain SX13, strain QK8 has fewer repetitive elements, including the major transposable element (TE) types (Table S3). 

#### 2.2.2. Gene Prediction and Annotation

The assembled genomes of strains QK8 and SX13 were 24,778,044 bp and 26,605,002 bp in length (average length: 1557 bp and 1598 bp), respectively. Moreover, 15,917 and 16,650 gene models were predicted for strains QK8 and SX13, respectively, accounting for 48.39% and 48.22% of their genome sizes. The protein-coding genes of strains QK8 and SX13 were functionally annotated through different databases, with 15,874 (99.73%) and 16,384 (98.40%) genes homologous to the NCBI Non-Redundant Protein Sequence (NR) database, 15,595 (97.98%) and 15,861 (95.26%) genes to the Kyoto Encyclopedia of Genes and Genomes (KEGG) database, 5540 (34.81%) and 7513 (45.12%) genes to the Gene Ontology (GO) database, 7045 (44.26%) and 7395 (44.41%) genes to the Eukaryotic Orthologous Groups (KOG) database, 924 (5.81%) and 759 (4.74%) genes to the Pathogen–Host Interactions (PHI) database, 156 (0.98%) and 152 (0.91%) genes to the cytochrome P450 monooxygenase (P450) database, 1680 (10.55%) and 1880 (11.29%) genes to the Carbohydrate-active Enzymes (CAZy) database, and 11,770 (73.95%) and 12,267 (73.68%) genes to the Protein families (Pfam) database, respectively (Table 2). 

**Figure 2:**
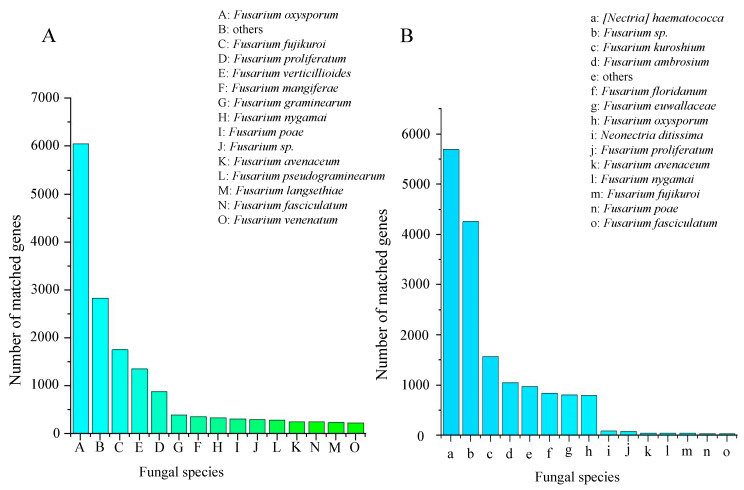
Results of the NR annotation results for strains QK8 (**A**) and SX13 (**B**) (15 strains).

In the NR database, annotated genomic hypothetical proteins accounted for 52.63% of the total genes in strain QK8 and 78.79% of the total genes in strain SX13. Moreover, of the genes in strains QK8 and SX13, 30.28% and 18.49% were annotated as specific functional proteins, respectively (Table 3). The best matching results was between strain SX13 and *Nectria haematococca* (5695), accounting for 34.76% of the total NR prediction motifs, indicating a close genetic relationship. This matching result was followed by *Fusarium* sp. (4257) and *F. kuroshium* (1572) (Figure 2B). Strain QK8 and *F. oxysporum* (6055) had the best matching results, indicating a close genetic relationship. This matching result was followed by *F. fujikuroi* (1758) and *F. proliferatum* (1356) (Figure 2A). Thirteen of the top 14 strains with the highest matching results with strain QK8 belonged to the *Fusarium* spp. population, with the matching results much higher than that of other strains. Among the predicted results, fewer genes of strain SX13 were predicted to be specific functional proteins compared to strain QK8 (Tables S4 and S5). The matching result between strain QK8 and *F. oxysporum* is the best, indicating that the two species are closely related.

Functional classification of strains QK8 and SX13 genes by the KOG database counted 6201 and 6509 genes, respectively (Tables S6 and S7). Except for some genes with unknown functions and the general function of “poorly characterized”, the gene category with the highest proportion of the genome of the two strains was related to metabolism. The main categories included “Carbohydrate transport and metabolism,” “Amino acid transport and metabolism,” and “Secondary metabolites biosynthesis, transport, and catabolism.” The second highest category was “Cellular processes and signaling”. The groups with the highest content were “Intracellular trafficking, secretion, vesicular transport,” “Posttranslational modification,” and “Signal transduction mechanisms.” Relatively few genes were in the information storage and processing class and were more evenly distributed in “Translation, ribosomal structure, and biogenesis,” “RNA processing and modification,” “Transcription and Replication,” and “Recombination and repair.” 

**Figure 3:**
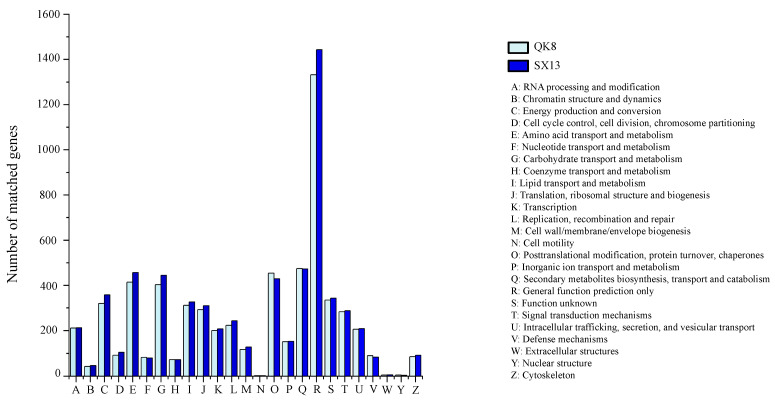
KOG functional classification of strains QK8 and SX13.

The most abundant genes for strains QK8 and SX13 were those involved in “General function” (1332 and 1443), followed by “Secondary metabolites biosynthesis, transport, and catabolism” (474 and 472), “Posttranslational modification, protein turnover, chaperones” (454 and 429), and “Amino acid transport and metabolism” (414 and 456) (Figure 3). The latter group of genes was related to protein transport or energy metabolism. Compared with strain QK8, strain SX13 had more of these genes, and the number of each gene was higher than that of strain QK8.

**Figure 4:**
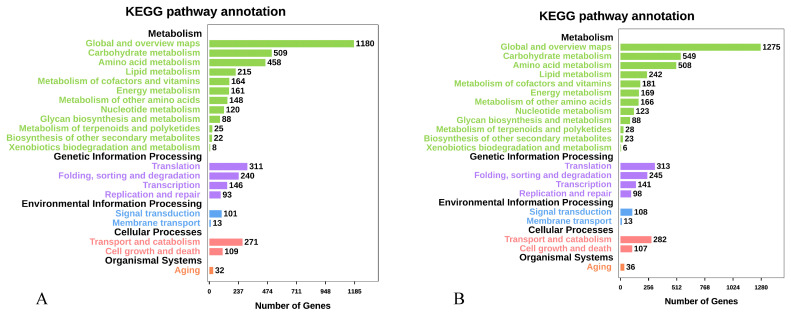
KEGG pathway annotation of strains QK8 (**A**) and SX13 (**B**).

The results of the KEGG database annotation showed that 15,595 and 15,861 gene models were annotated for strains QK8 and SX13, respectively, accounting for 4.28% and 5.40% of their genomes (Tables S8 and S9). The metabolism class had the largest number of genes from strains QK8 and SX13. Among the gene functional categories of strains QK8 and SX13, some categories related to metabolism and genetic information processing were highly enriched, including “Global and overview maps” (1180 and 1275), “Carbohydrate metabolism” (509 and 549), “Amino acid metabolism” (458 and 508), “Translation and Folding” (311 and 313), and “Sorting and degradation” (240 and 245) among others (Figure 4A,B). In KEGG annotation results, the number of genes of strain SX13 was higher than that of strain QK8 in most categories; however, the distribution of their genes was very similar, with almost no difference in the number of genes in some categories.

**Figure 5:**
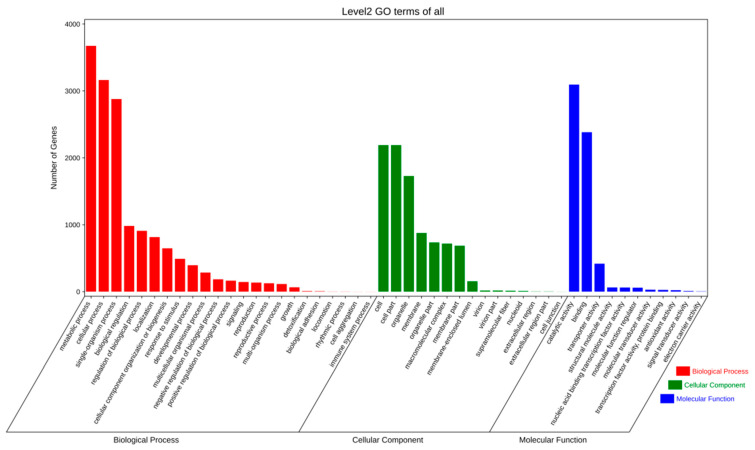
GO functional annotation of strain QK8.

**Figure 6:**
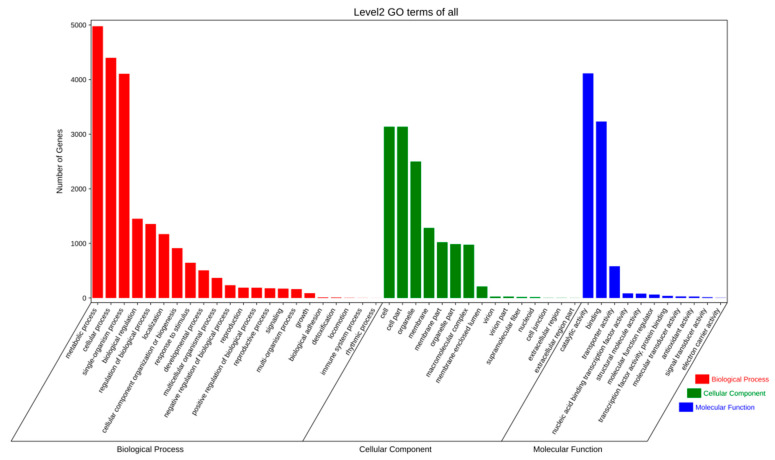
GO functional annotation of strain SX13.

Through GO functional annotation and classification analysis, we found 5540 and 7513 annotated proteins models and functionally assigned proteins for strains QK8 and SX13, respectively, accounting for 14.31% and 43.53% of their genomes (Tables S10 and S11). The quantitative distribution of GO class genes in strains QK8 and SX13 was very similar, wherein the largest number of GO terms was “metabolic process,” “cellular process,” “sing-organism process,” “cell,” “cell part,” “organelle,” “catalytic activity,” and “binding.” The number of genes was much higher than that of other GO terms (Figure 5 and Figure 6). 

#### 2.2.3. CAZyme Annotation

Strains QK8 and SX13 contained 41 and 43 polysaccharide lyases (PLs), 198 and 263 auxiliary activities (AAs), 649 and 653 glycoside hydrolases (GHs), 405 and 444 glycosyl transferases (GTs), 140 and 138 carbohydrate esterases (CEs), and 247 and 259 carbohydrate-binding modules (CBMs), respectively (Table 4). Strains QK8 and SX13 had 830 and 834 predicted CE, GH, and PL CAZyme genes, accounting for 49.40% and 46.33% of the total predicted CAZymes, respectively.

In the carbohydrate annotation of strains Fo47 and 160,527 (which is closely related to strain QK8), 1679 and 1812 genes were annotated, respectively (Table S12). Compared to the carbohydrate annotation results among the three strains, there was little difference in the number of genes in the CBM and CE classes. However, there were significant differences in other classes, particularly in the GH and GT classes, which were associated with pathogenicity. In particular, there were significant differences between strains QK8 and 160527. For strain SX13, the closely related strains FSSC 5 MPI-SDFR-AT-0091 and CRI24-3 were annotated with 1797 and 1727 genes, respectively (Table S13). The main differences among the three strains were found in the AA, GH, and GT classes. For the GH class, strain SX13 was closer in gene number to strain FSSC 5 MPI-SDFR-AT-0091. For the GT class, there were significant differences between strain SX13 and the two other strains. There were no significant differences among the three strains for the other classes.

Among the genes of the GH family encoded by *F. oxysporum* and *F. solani*, the most abundant QK8 genes were GH43 (62), GH16 (50), GH3 (42), GH18 (62), and GH5 (25); the most abundant SX13 genes were GH3 (54), GH43 (61), GH18 (52), GH16 (46), and GH5 (20). It is worth noting that the content of key pathogenic GHs (GH18, GH16) of strain QK8 is significantly higher than that of strain SX13. The three most abundant CE families of the two strains were CE1, CE4, and CE6. The number of these genes in the QK8 strain was higher than that in the SX13 strain, except for CE6. The most abundant families of polysaccharide lyases in these two strains were PL1 and PL4, and the number of these two types of genes in strain QK8 was higher than that in strain SX13. The most abundant GT families of the two strains were GT2 and GT4, and their contents were much higher than those of other types of GT, followed by GT1 (Tables S16 and S17). 

#### 2.2.4. Pathogenicity-Related Gene Analysis

**Figure 7:**
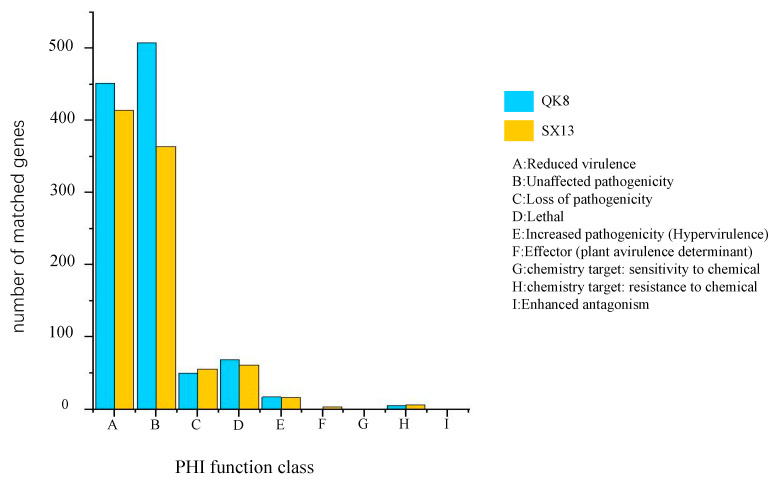
PHI functional annotation of strains QK8 and SX13.

We annotated the PHI genes and analyzed the results statistically (homology ≥ 70%; score < 1 × 10^20^). Here, 1007 and 836 PHI genes were annotated in strain QK8 and strain SX13, respectively, accounting for 6.33% and 5.02% of the total predicted genes (Tables S18 and S19). The predicted number of PHI categories in strain QK8 was sorted from high to low as “Unaffected pathogenicity” (508, 50.45%), “Reduced virulence” (451, 44.79%), “Lethal” (69, 6.85%), “Loss of pathogenicity” (50, 4.97%), “Increased pathogenicity” (Hypervirulence) (17, 1.69%), “Chemistry target: resistance to chemical” (5, 0.50%), “Effector” (plant avirulence determinant) (1, 0.10%) and “Chemistry target: sensitivity to chemical” (1, 0.10%). For strain SX13, the predicted PHI categories were “Reduced virulence” (414, 49.52%), “Unaffected pathogenicity” (364, 43.54%), “Lethal” (61, 7.30%), “Loss of pathogenicity” (56, 6.70%), “Increased pathogenicity” (Hypervirulence) (16, 1.91%), “Chemistry target: resistance to chemical” (6, 0.71%), “Effector” (plant avirulence determinant) (3, 0.36%), and “Chemistry target: sensitivity to chemical” (1, 0.12%). No “Effect factor” (enhanced antagonism) was found in the two strains screened under the set statistical criteria (Figure 7).

The genomes of strains closely related to strains QK8 and SX13 were selected for PHI annotation and compared. In the PHI database, strains Fo47 and 160527 were annotated with 669 and 651 genes, respectively (Table S14). Both strains not only had far fewer annotated genes but also fewer genes in all PHI classes than strain QK8 (except for the PHI class, which was absent from all three strains). Both *F. solani* strains FSSC 5 MPI-SDFR-AT-0091 and CRI 24-3 had 837 genes annotated in PHI database (Table S15). There was little difference in PHI genes among strains SX13, FSSC 5 MPI-SDFR-AT-0091, and CRI 24-3, as well as in most PHI classes. For example, the number of genes in strains FSSC 5 MPI-SDFR-AT-0091, CRI 24-3, and SX13 was 15, 16, and 16, respectively. Within the “attenuated” category, the largest differences in the number of genes were found in strains FSSC 5 MPI-SDFR-AT-0091(417), CRI 24-3 (427), and SX13 (414), with the other PHI categories having no more than six genes.

**Figure 8:**
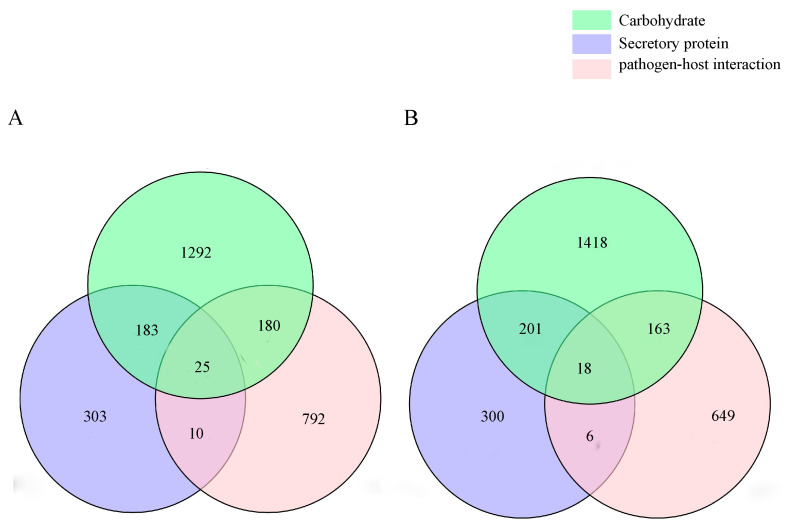
Relationships between genes in the three annotated plates of strains QK8 (**A**) and SX13 (**B**).

Using SignalP and PredGPI, we predicted that strains QK8 and SX13 had 521 and 525 possible secretory proteins, respectively. The possible effector proteins of these predicted secreted proteins we predicted (amino acid length ≤ 300; CYs ≥ 4). Both QK8 and SX13 had 167 candidate effector proteins, accounting for 31.87% and 31.81% of their secreted proteins, respectively (Table S20). It is worth noting that we used different software for annotation analysis, and the annotation results are more convincing. Strains QK8 and SX13 contained 11,770 and 12,267 genes containing Pfam domains, respectively, and 156 and 152 genes containing cytochrome P450 domains, respectively (Tables S21 and S22). Through analysis, we found that the secretory protein genes of strain QK8 contained 208 genes of CAZymes, 35 genes of PHI, 205 common genes of PHI and CAZyme, and 25 genes in all three databases. In strain SX13, there were 24 overlapping genes in the PHI and secretory protein databases, 24 overlapping genes in the PHI and secretory protein databases, 219 overlapping genes in the CAZyme and secretory protein databases, 181 overlapping genes in the CAZyme and PHI databases, and 18 overlapping genes in all three databases (Figure 8A,B). The IDs of these overlapping genes are listed in Tables S23 and S24. 

#### 2.2.5. Phylogenetic Analysis

**Figure 9:**
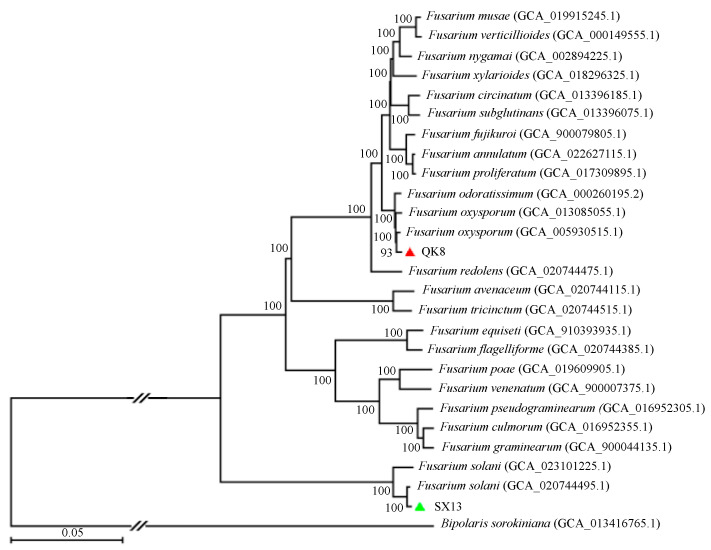
The genomic phylogenetic tree of strains QK8 and SX13 alongside 25 other species of fungi. There are 1739 single-copy homologous genes. The bootstrap value represents the percentage of related strains clustered together in the phylogenetic tree. The information in parentheses indicates the genome assembly fungal assembly, the triangle symbols indicate the location of strains *F. oxysporum* strain QK8 (redΔ) and strain *F. solan* SX13 (greenΔ), and the root is *Bipolaris sorokiniana*.

Strains QK8 and SX13 along with 24 other *Fusarium* spp. (Table S25) were used to construct a phylogenetic tree (Figure 9), with *Bipolaris sorokiniana* as an outgroup to root the tree. The phylogenetic tree was constructed using 1739 single-copy orthologous genes. All fungi had a good support rate. Strain QK8 and *F. oxysporum* (genome assembly: GCA_005930515.1) were clustered together with a support rate of 93%, indicating a close genetic relation. Strain SX13 and *F. solani* (genome assembly: GCA_020744495.1) were clustered on one branch with a support rate of 100%, indicating a close genetic relation. 

## 3. Discussion

In this study, by comparing the biological characteristics of the two isolated strains, the results indicated that the pathogen can grow when the air temperature is above 10 °C; the suitable conditions for QK8 and SX13 pathogen development are 28 °C or 30 °C at pH 7 or 9, respectively. Therefore, brown rot is more likely to break out in neutral or alkaline soils under high temperatures and humidity in summer. Hence, these parameters can be used to estimate the control time.

Studies have shown that methanolic extracts from *Artemisia annua* leaves have a significant inhibitory effect on the growth of *F. oxysporum* and *F. solani*, which cause root rot in *Panax notoginseng* [27]. Furthermore, *Bacillus subtilis* HSY21 could inhibit soybean root rot caused by *F. oxysporum* [28]. Prochloraz and other commercial fungicides significantly inhibit the genus *Fusarium* that causes *Allium cepa* basal rot disease [29]. Propiconazole + prochloraz could effectively inhibit the growth of *Fusarium* species involved in garlic dry rot [30]. In our indoor toxicity test, oxime tebuconazole, tebuconazole, and tetramycin had a good antifungal effect on the two *Fusarium* strains; their EC_50_ values ranged from 0.0836 to 0.7220 μg/mL, which could be used as the first choice for field control experiments. Other fungicides with good antifungal effect included pentachloronitrobenzene, thiram·carbendazim, and imazalil, with EC_50_ values ranging from 1.3088 to 4.0920 μg/mL. These biological studies on diseases caused by *Fusarium* species have positive implications for the prevention and control of these diseases and can provide theoretical guidance and support for disease prevention and control. Differences in soil nutrients can affect the yield of black morel [31], and the diversity and homogeneity of soil microorganisms can affect morel production [32]. Multi-omic analyses have revealed the nutrient acquisition and transfer of black morel [33]. The characteristics of soil microbiota can be used to predict the potential of *Fusarium* wilt occurrence [34]. These findings provide new ideas for developing effective disease control measures and for further research.

Genome sequencing provides insight into the pathogenic mechanism of pathogenic organisms. In this study, the genomes of *G. elata* brown rot isolate *F. solani* strain SX13 and *F. oxysporum* strain QK8 were sequenced by Illumina and PacBio sequencing, and high-quality genomic sequences were obtained. The genome size of strain QK8 is about 51,204,719 bp, with 14.94% repeat loci consisting of 21 scaffolds and 15,917 genes. Through annotation analysis, 5540 genes were assigned to GO and 15,595 genes to KEGG. The genome size of strain SX13 is 55,171,989 bp, with 17.23% repeat loci consisting of 21 scaffolds and 16,650 genes. Through annotation analysis, 7513 genes were assigned to GO and 15,861 genes to KEGG. There were significant differences in genomes among *F. oxysporum* strain QK8 and strains Fo47 [22] and 160527 [35], while *F. solani* strain SX13 was similar to strains FSSC 5 MPI-SDFR-AT-0091 [19] and CRI 24-3 [21]. These results provide a useful resource for follow-up experiments.

Phenotypic characterization and phylogenetic analysis based on internal transcribed spacer (ITS) can determine the taxonomic status of strains because of the high conservation, interspecific variation, and high availability of ITS [36]. Some other gene markers are also used in taxonomic studies, and translation elongation factor 1-α (TEF 1-α) is used to distinguish among species [37]. Moreover, methods such as genome sequencing have greatly improved the study of taxonomy, genetic diversity, and pathogenic mechanisms [38,39,40]. Compared with the phylogenetic research based on ITS, genomics-based phylogenetic studies can provide more information and can support the identification of strains (as in our research). Combining these two methods will provide a more comprehensive understanding of strains. Genomic approaches provide an excellent opportunity to describe species development and understand the underlying mechanisms [41].

The cell wall is a very important line of defense; therefore, cell wall-degrading enzymes play a key role in destroying the plant cell wall and enabling pathogenic fungi invasion. The complexity and diversity of the cell wall are reflected by the diversity of CAZymes that facilitate the invasion of pathogenic fungi [42,43]. Here, 1680 coding genes were predicted in strain QK8’s genome and 1800 in strain SX13’s genome, all of which were assigned to six types of CAZymes (PLs, AAs, GHs, GTs, CBMs, and CEs). CE, GH, and PL class CAZymes are called cell wall-degrading enzymes and may play a key role in pathogenicity by participating in the process of cell wall degradation [44]. The two strains were rich in GH (e.g., GH3 and GH43) and GT (e.g., GT2 and GT4) class. These abundant genes are closely related to the cell wall. Among them, the abundance of GH class genes was closely related to the effective degradation of chitinase, cellulase, and hemicellulase. GT class genes play an important role in glycosylation, cell wall biosynthesis, chitin synthesis, and various metabolic processes [45,46]. Moreover, rich PL (e.g., PL1 and PL4) class genes may have pectin-specific activity [47]. It is worth noting that the GH class CAZymes in both *Fusarium* strains contain a large number of the total predicted CAZymes genes, similar to other *Fusarium* spp. [19,48]. This indicates that the GH family plays an important role in *Fusarium* pathogenicity. Moreover, there may be many proteolytic enzymes that interact with the *Fusarium* host. Other studies have shown that when CAZymes enter plants, they can cause a plant defense response [49] and induce a plant immune response [50].

The effect factor determines the virulence of the pathogenic fungi to a large extent. It plays an important role in host invasion and disease [51]. Pathogenic fungi can optimize their effectors to adapt to the host and secrete proteins for host surface colonization during infection [52,53]. Genome annotation analysis predicted that strains QK8 and SX13 contain 167 candidate effector proteins, accounting for 31.87% and 31.81% of the total secreted proteins, respectively. PHI annotation analysis showed that 998 and 825 candidate pathogenicity-related proteins were annotated for strains QK8 and SX13, respectively. It is worth noting that overlaps exist between secretory protein injection genes, CAZymes genes, and PHI genes in strains QK8 and SX13, and the three databases contain 15 and 13 genes, respectively. To some extent, the pathogenicity is very likely to be related to these genes. These findings may be helpful for understanding the interaction between the two *Fusarium* strains and *G. elata*.

## 4. Materials and Methods

### 4.1. Source of Pathogenic Fungi of G. elata Brown Rot

The *G. elata* brown rot isolates were numbered QK8 and SX13; previous studies have shown them to be *F. oxysporum* and *F. solani*, respectively [11,12]. The strains are stored in a frozen tube containing 15% glycerol in a refrigerator at 4 °C in the Key Laboratory of Agricultural Microbiology at Guizhou University. 

### 4.2. Determination of Biological Characteristics

For pH determination, 0.1% HCL and 0.1% NaCl were used to screen the optimum pH for fungal growth. Cha’s medium (Czapek; Sigma Aldrich, St. Louis, MI, USA) was used as a basic medium supplemented with different carbon and nitrogen sources. This was used to screen the carbon and nitrogen sources most suitable for the growth of the two strains. Seven media were used for optimal culture medium screening: PDA, OA, NA, BRAM, SNA, CDM, and MEA. The effect of light on fungal growth was tested by changing the light conditions. The optimal growth (culturing strains in incubators with different temperatures) and lethal temperatures (heating in a water bath for a certain period and then culturing) were tested (Table S26). Seventeen fungicides were used to determine the antifungal susceptibility of the two strains (Table S27). The mycelium growth rate method was used to determine the effect of the tested agents on the growth of the strains. The fungicide concentration was set to 2.5 × 10^3^, 1.25 × 10^3^, 6.3 × 10^2^, 3.1 × 10^2^, 1.6 × 10^2^, 8 × 10^1^, 4 × 10^1^, 2 × 10^1^, and 1 × 10^1^ μg/mL. Sterile water was used as the control.

### 4.3. Draft Genome Sequencing, Assembly, and Repetitive Sequences Analysis

Strains QK8 and SX13 were cultured in potato dextrose broth on a shaker at 25 °C and 210 rpm for 5 days. The hyphae were collected and genomic DNA extracted using the Fungal Genome DNA extraction kit (Novogene Technology, Beijing, China). Genome sequencing was carried out by Novogene Technology on an Illumina HiSeq 2000 platform; a DNA library was then established. Trimmomatic_v0.32 [54] was used to filter out low-quality reads with a total length < 75 bp to obtain high-quality genomic data. Concurrently, genome sequencing was carried out on a PacBio Sequel sequencing platform. After the genomic DNA was fragmented, fragments > 20 Kb were recovered by the BluePippin system to prepare a DNA library. Next, SMRTlink_v5.0 (Pacific Biosciences Technology, Beijing, China) was used to filter the output results to remove low-quality reads, to obtain effective data.

SOAPec_v2.01 [55] was used to evaluate the genome size of the two strains from the two sequencing datasets under the parameter “Genome size = kmer_Number/Peak_Depth”. SOAPdenovo_v2.04 [55] and SSPACE_v3.0 [56] were used to assemble the Illumina sequencing data, and GapCloser_v1.12 [55] was used to fill gaps in the assembly results. HGAP4 [57] was used to assemble the PacBio sequencing data, Canu_v1.5 [58] and MECAT_v1.3 [59] were used to detect the assembly results, and plion_v1.22 [60], combined with double terminal reading data with Illumina, was used to modify the splicing results to improve the accuracy of the single bases. DBG2OLC [61] was used to mix and assemble the Illumina and PacBio sequencing data to verify the difference between pure Illumina sequencing data splicing and pure PacBio sequencing data splicing. RepeatModeler (http://www.repeatmasker.org/RepeatModeler.html, accessed on 8 July 2022), LTR-FINDER [62], and LTR_retriever [63] were used to identify the repetitive sequences of the genomic components.

### 4.4. Gene Prediction and Annotation

Three prediction methods are used for gene prediction: prediction based on transcriptome data, de novo prediction, and homologous protein annotation prediction. Here, we used de novo prediction and homologous protein annotation prediction. Genes of the two strains were predicted using Augustus [64], GeneMark+ES [65], and SNAP [66]. The protein-coding regions were predicted by homology. The published protein sequences of *F. oxysporum* and *F. solani* were mapped to the assembled genomes of strain QK8 and SX13, and genomic prediction based on homology was carried out by Exonerate [67]. The final gene model was obtained by EvidenceModeler [68] software integration. The predicted genes were annotated by BMKCloud [69], and the protein sequences were uploaded to the functional annotation plate of https://international.biocloud.net/gene (accessed on 8 July 2022). The databases of nr_vs_GO, KEGG, CogSwissProt, TrEMBL, KOG, and Pfam were annotated, and the parameters were ‘fungi’ or ‘total’. The predicted genes were annotated by CAZy (carbohydrate-active enzymes) using dbCAN [70] software and the protein sequences were uploaded to https://bcb.unl.edu/dbCAN2/blast.php (accessed on 8 July 2022) for online annotation. The OmicShare tools with default parameters were used for annotation analysis (https://www.omicshare.com/tools, accessed on 8 July 2022). The TBtools [71] software was used to predict Pfam domains. The PHI genes were annotated through the http://www.phi-base.org/website (accessed on 8 July 2022) [72]. KEGG and GO analyses were performed using the OmicShare tools.

### 4.5. Analysis of Secretory and Effector Proteins

The annotation of secreting proteins was divided into the following steps. First, SignalP_v6.0 Server [73] was used to predict signal peptides; then, TMHMM Server_v1.0.10 [74] and phobius_v1.01 [75] were used to predict transmembrane domains. Then, WoLF PSORT [76] and ProtComp_v9.0 [77] were used to reveal the subcellular localization of the extracellular proteins, and finally, PredGPI [78] was used to remove GPI-anchor proteins. The output of this workflow was the candidate secretory proteins. The candidate secretory proteins were screened according to the criteria of amino acid (AA) size (≤300 AA) and cysteine richness (≥4 cysteine residues). Cysteine residues were screened using the SnapGene_v6.0 (GSLBiotech) software.

### 4.6. Phylogeny and Homology Analyses

Lineal homologous gene and phylogenetic analyses were conducted using OrthoMCL [79]; all-versus-all BLASTP was used to identify homologous groups (E-value ≤ 1 × 10^15^, coverage ≥ 50%) of protein datasets, using *Saccharomyces cerevisiae* as a reference for draft genome replication. The Perl script (command line parameter of Gblocks: Gblocks protein.fasta-b4 = 5-b5 = h.) was used to extract the single copy directly to the homologous sequence. MAFFT [80] was used to calibrate the extracted data, in which the poorly aligned part of the tandem sequence was eliminated by Gblocks [81]. RAxML [82] was used to reconstruct the maximum likelihood phylogeny.

## 5. Conclusions

In this study, we performed whole-genome sequencing analysis and annotation on these two strains of *Fusarium* to study the pathogenic mechanisms of these two strains of *Fusarium* and predict the pathogenic genes from the genome level. Genomic information on two fungal strains deepens our understanding of two *Fusarium* strains, accelerates their future functional studies, and contributes to the control of *G. elata* brown rot. The *G. elata* brown rot fungus was characterized using a biological characterization approach. The optimum temperature and pH data of two *Fusarium* strains can be used as a basis for the prevention and control of brown rot, while the indoor virulence tests provide a basis for the selection of agents to control brown rot. 

## Figures and Tables

**Table 1:** General genomic features of the two *Fusarium* strains.

Scaffold Characteristics	QK8	SX13
Total counts of contig sequences	21	21
N50 Length (bp)	4,657,513	3,724,189
N90 Length (bp)	2,323,049	1,334,751
Longest Length (bp)	6,849,339	6,503,946
Shortest Length (bp)	106,531	704,098
tRNA	282	275
rRNA	63	70
Average Length (bp)	2,438,320	2,627,238
Genome coverage (%)	98.04	97.91
GC content (%)	47.21	50.61
Total Size (bp)	51,204,719	55,171,989
Gene Characteristics		
Number of genes	15,917	16,650
Exon average length (bp)	1557	1598
Genome GC percent (%)	47.21%	50.61
Exon Gene GC percent (%)	51.43	54.91
Total Size (bp)	24,778,044	26,605,002

**Table 2:** Annotated summary table of two *Fusarium* strains QK8 and SX13.

Annotation Database	Number of Genes	
	QK8	SX13
Carbohydrate-active Enzymes Database (CAZy)	1680	1800
Kyoto Encyclopedia of Genes and Genomes (KEGG)	15,595	15,861
Eukaryotic Orthologous Groups (KOG)	7045	7395
Gene Ontology (GO)	5540	7513
Cytochrome P450 monooxygenase (P450)	156	152
Pathogen–Host Interactions Database (PHI)	1007	836
NCBI Non-Redundant Protein Sequence Database (NR)	15,877	16,384
Protein families database (Pfam)	11,770	12,267

**Table 3:** Results of the gene function annotation results of strains QK8 and SX13.

ID	Uncharacterized Proteins	Hypothetical Proteins	Functional Genes	Total
QK8	2678	8377	4819	15,874
SX13	186	13,119	3079	16,384

**Table 4:** Results of the CAZyme functional classification of strains QK8 and SX13.

Isolate	PLs	AAs	GHs	GTs	CEs	CBMs	Total
QK8	41	198	649	405	140	247	1680
SX13	43	263	653	444	138	259	1800

Abbreviations: PLs, Polysaccharide lyase; AAs, Auxiliary activities; GHs, Glycoside hydrolase; GTs, Glycosyl transferase; CEs, Carbohydrate esterase; CMBs, Carbohydrate-binding module.

## Data Availability

The assembly and sequence data of the two *Fusarium* strains QK8 and SX13 are available in NCBI BioProjects PRJNA903523 and PRJNA903524, respectively. Other data are available on request from the corresponding author.

## Supplementary Materials

The supporting information can be downloaded at: https://www.mdpi.com/article/10.3390/ijms24054545/s1.
