# Supplementary figures and images for: Draft Genome and Biological Characteristics of Fusarium solani and Fusarium oxysporum Causing Black Rot in Gastrodia elata
Source: Int J Mol Sci. 2023 Feb 25;24(5):4545. doi: 10.3390/ijms24054545 (PMC10003674; doi:10.3390/ijms24054545)

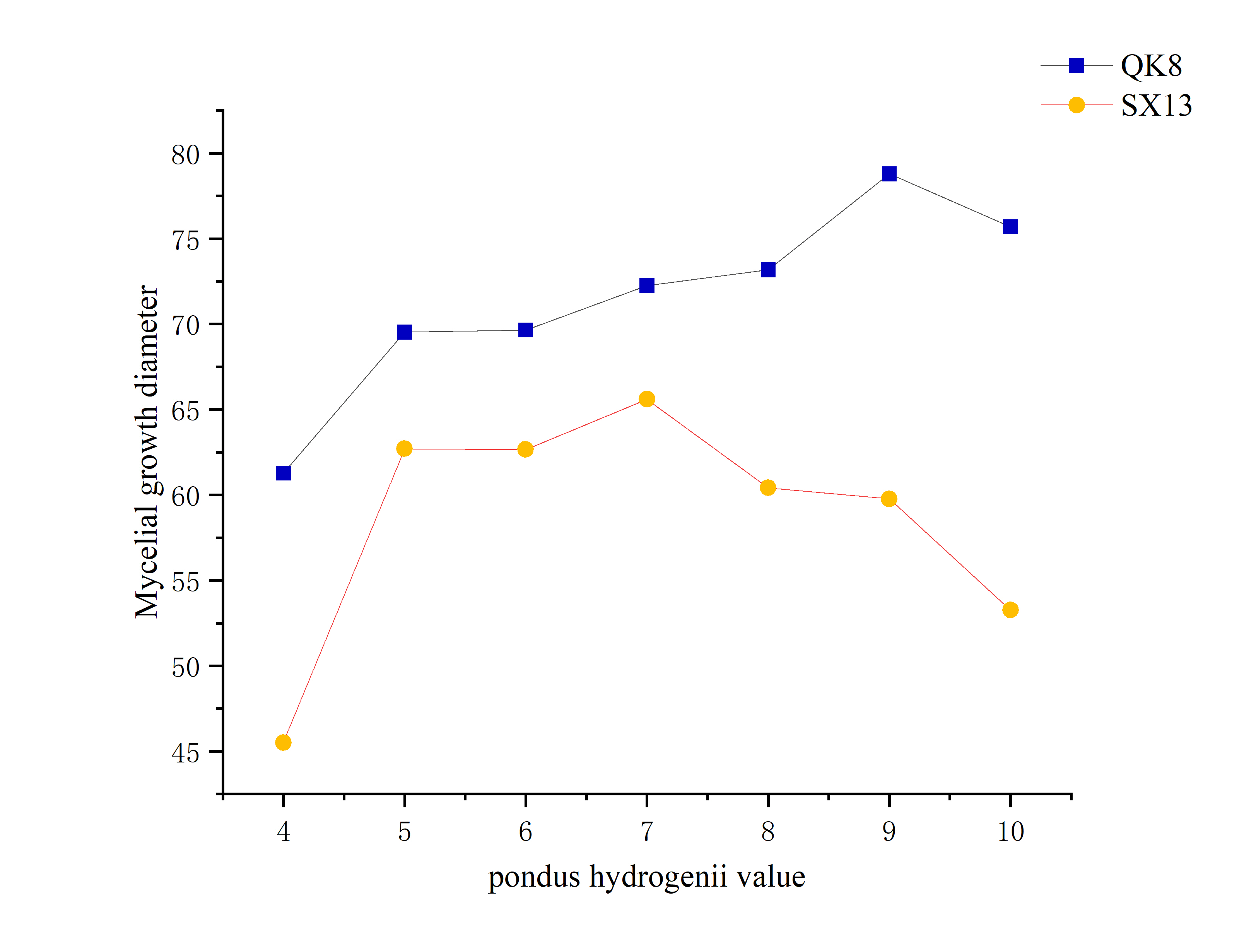

Supplement: Supplementary file 1 [file ijms-24-04545-s001.zip › Figure/Figure S1 Screening of the optimal PH value for the growth of strains QK8 and SX13.jpg]

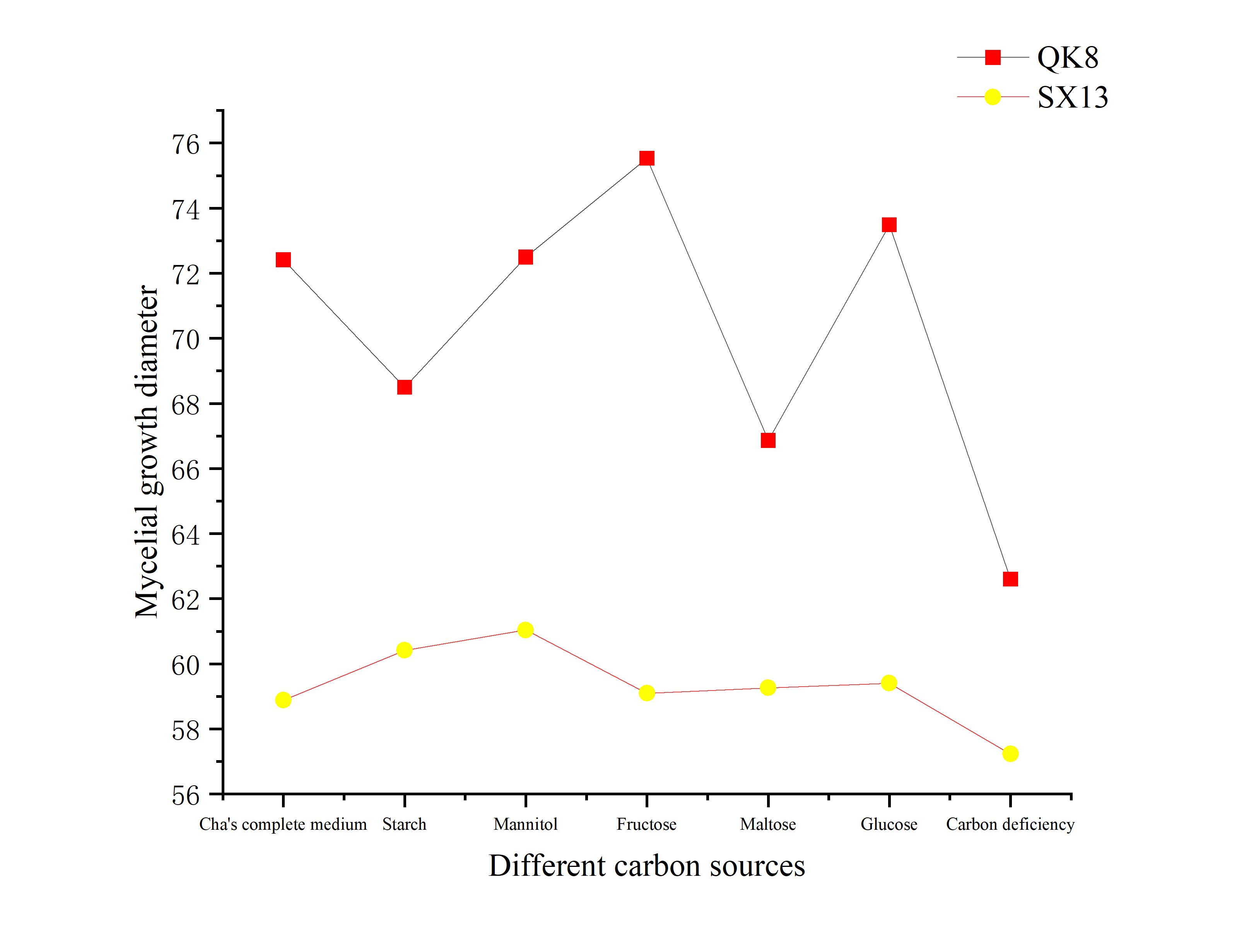

Supplement: Supplementary file 1 [file ijms-24-04545-s001.zip › Figure/Figure S2 Screening of the optimal carbon sources for the growth of strains QK8 and SX13.jpg]

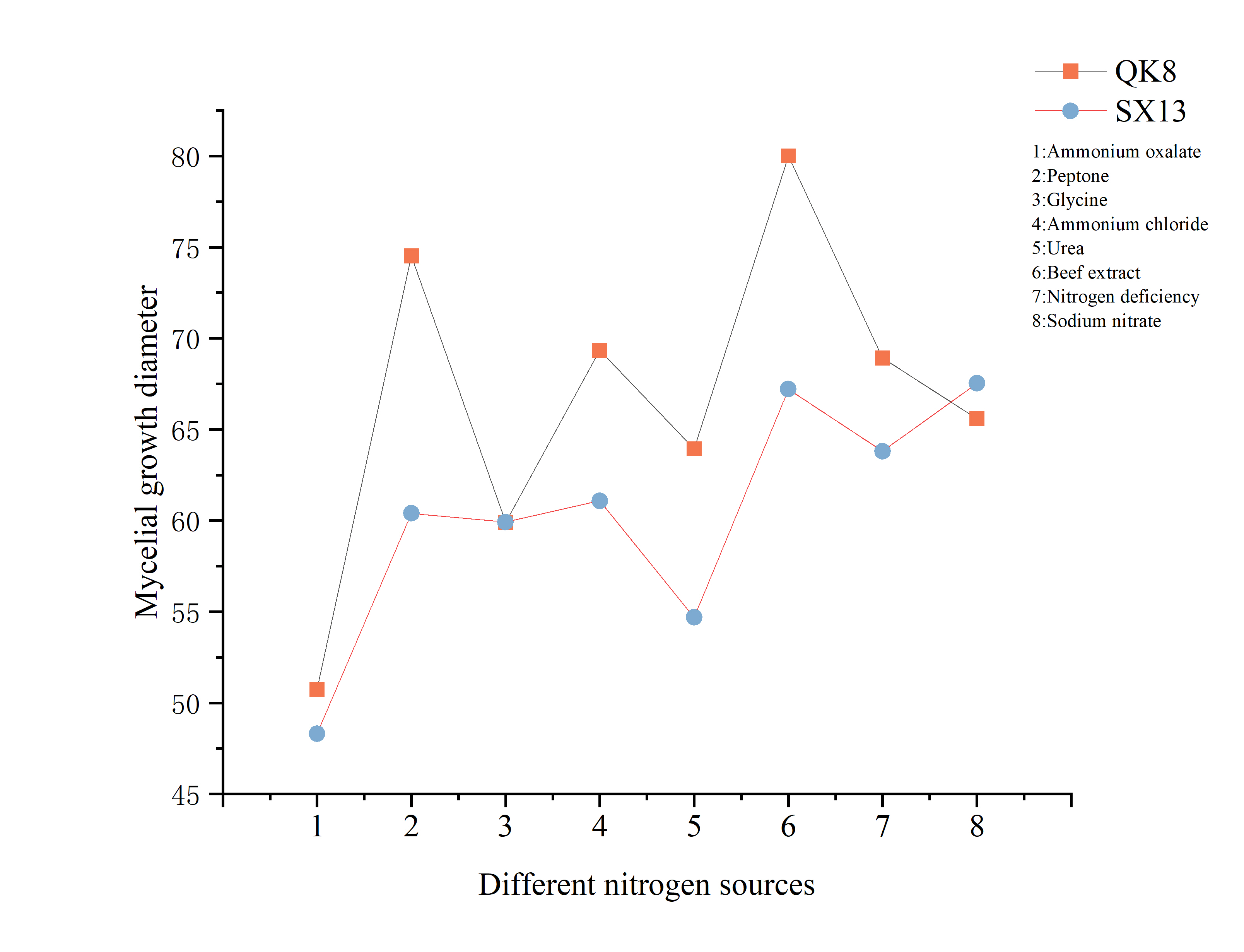

Supplement: Supplementary file 1 [file ijms-24-04545-s001.zip › Figure/Figure S3 Screening of the optimal nitrogen sources for the growth of strains QK8 and SX13.jpg]

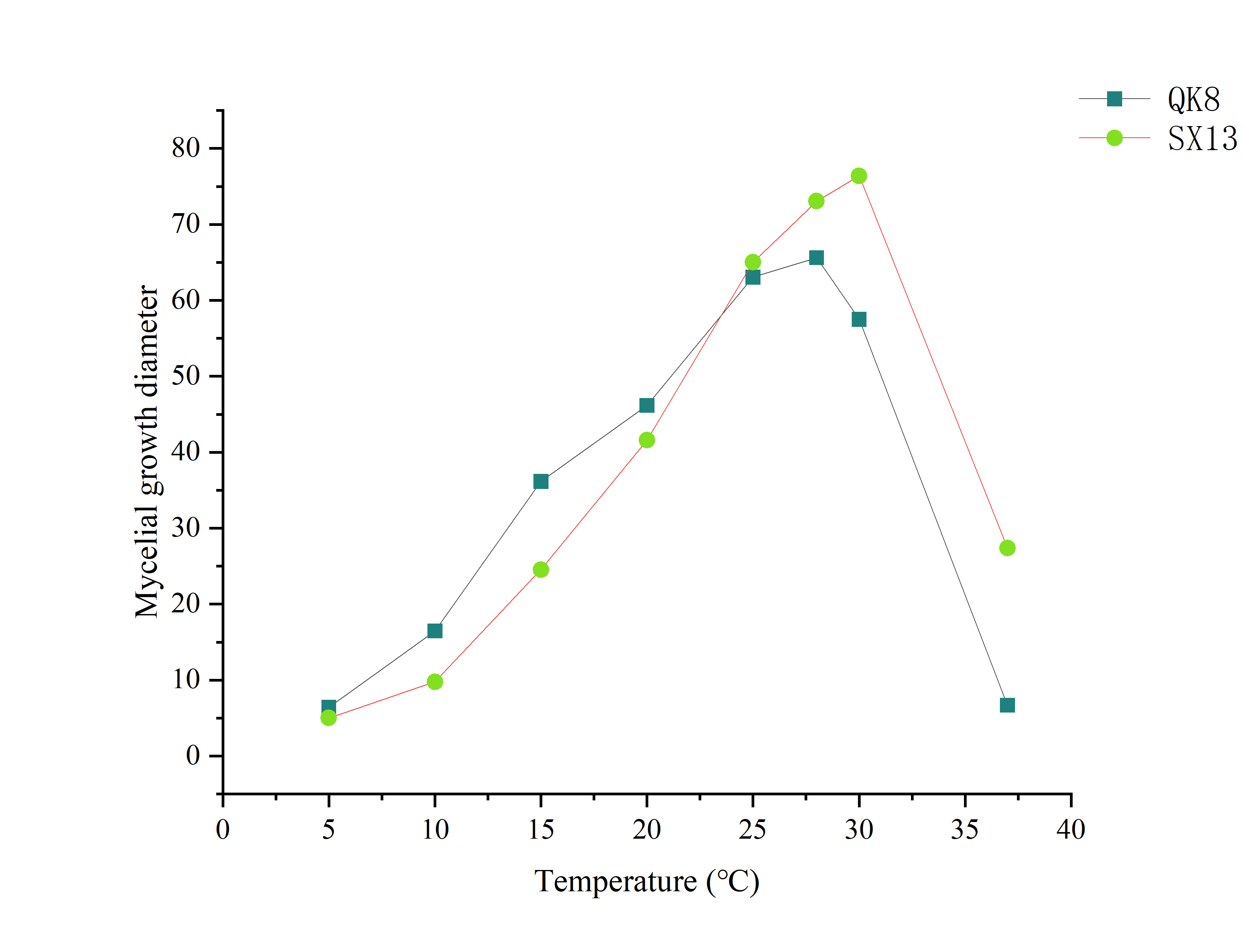

Supplement: Supplementary file 1 [file ijms-24-04545-s001.zip › Figure/Figure S4 Screening of the optimal temperature for the growth of strains QK8 and SX13.jpg]

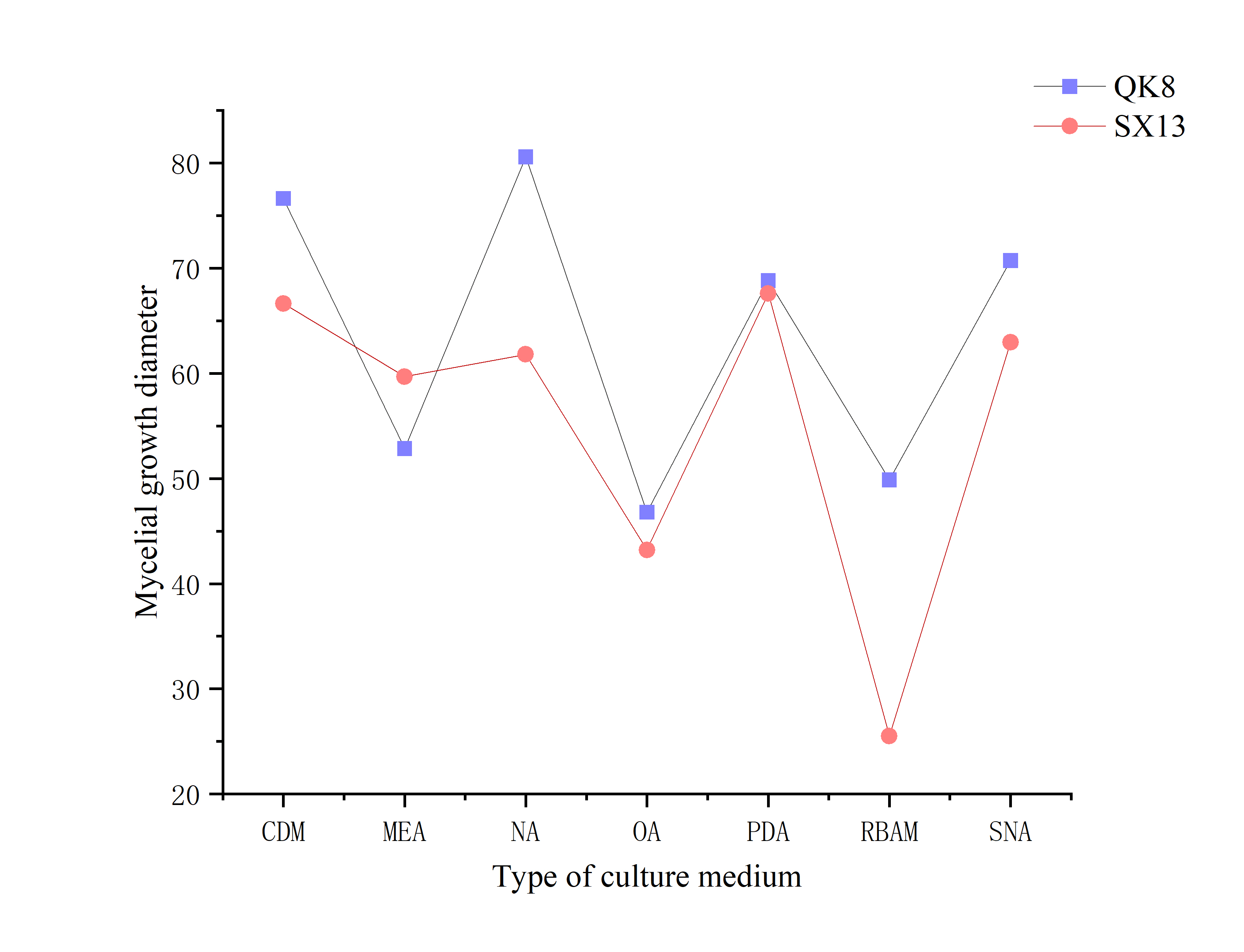

Supplement: Supplementary file 1 [file ijms-24-04545-s001.zip › Figure/Figure S5 Screening of the optimal culture medium for the growth of strains QK8 and SX13.jpg]

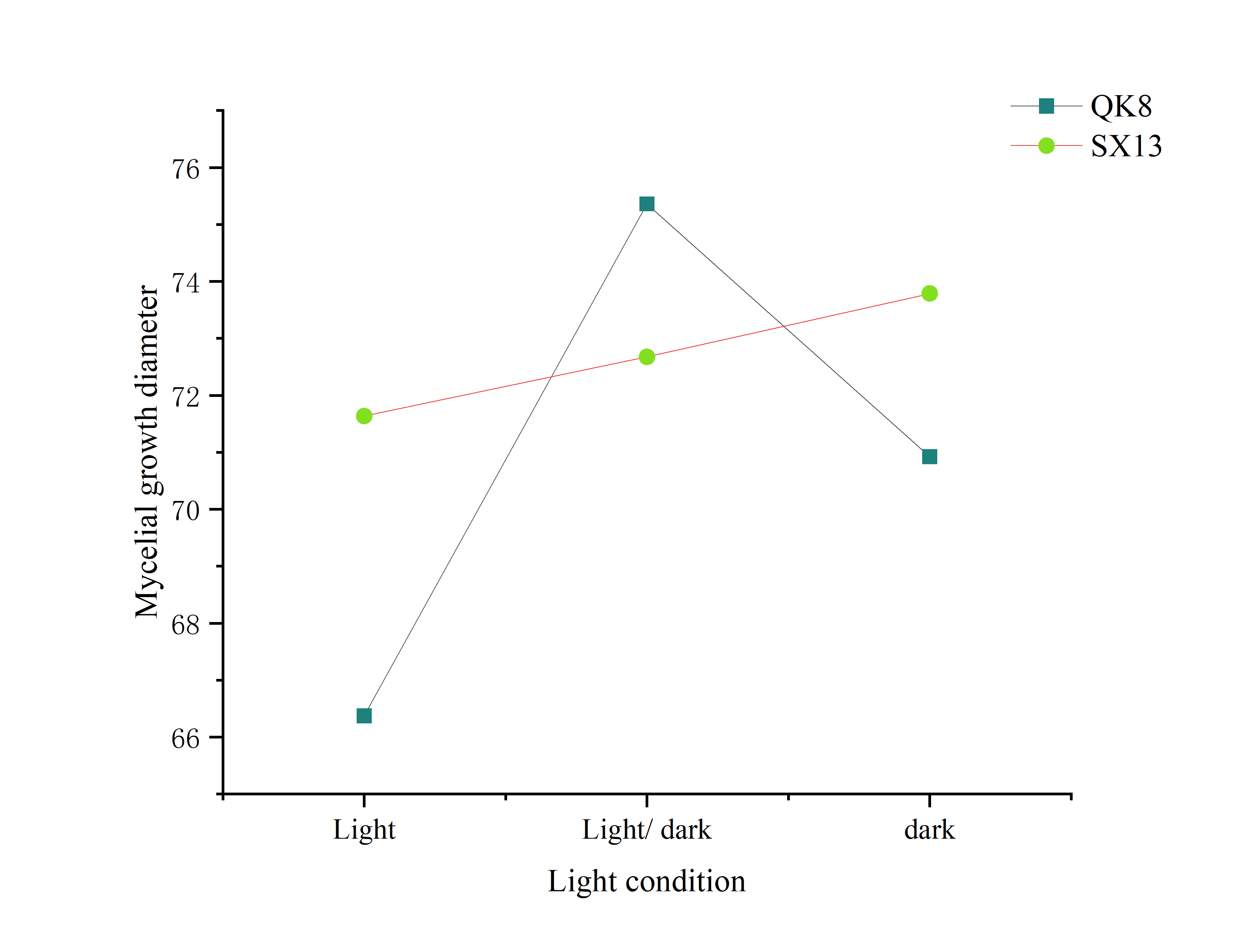

Supplement: Supplementary file 1 [file ijms-24-04545-s001.zip › Figure/Figure S6 Effect of light on the growth of strains QK8 and SX13.jpg]
